# Atmospheric Releases of Air-activated Radionuclides from Medical Cyclotrons: A Comparison among the Models Related to Population Doses

**DOI:** 10.1097/HP.0000000000002106

**Published:** 2026-02-13

**Authors:** Simone Manenti, Pietro Castellone, Flavia Groppi, Antonella del Vecchio, Riccardo Calandrino

**Affiliations:** 1 Department of Physics, University of Milan, Via Giovanni Celoria 16, I-20133 Milano, Italy; 2 Radiation Safety Officer, Cyclotron Dept, Clinica La Maddalena, Via Lorenzo Colli 312, I-90146, Palermo, Italy; 3 Medical Physics Department, Ospedale San Raffaele, Via Olgettina 60, I-20132 Milano, Italy.

**Keywords:** cyclotron, dose, population, emissions, atmospheric, neutron activation

## Abstract

A comparison among three models (Birattari Conic Plume, HotSpot, and NCRP 123 Gaussian distribution) has been carried out concerning the release of air-activated radionuclides during bombardment of a H_2_^18^O target in a medical cyclotron with two different proton energies: 10 MeV and 18 MeV. Under worst-case meteorological conditions, the diffusion of ^13^N, ^40^Cl, ^37^S, and ^41^Ar has been investigated. The total amount of induced activation (Bq) during different beam times has been calculated for each isotope, and the three models have been used to assess the dose to the residential population located 100 m from the release point, for a fixed beam current of 100 µA. The results demonstrate that a facility for medical isotope production, even under heavy workload (3,000 µA h^−1^ wk^−1^), will not lead to a significant increase in the dose to the surrounding population.

## INTRODUCTION

The topic of air activation in a medical cyclotron has been widely discussed by some authors in the past (Keshavkumar et al. 2017; Birattari et al. 1986, 1987; Cicoria et al. 2017; Infantino et al. 2015).

Several studies have addressed the production and dispersion of airborne radionuclides in medical cyclotron facilities, with a particular focus on ^41^Ar and other short-lived activation products. Infantino et al. (2015) combined experimental measurements and FLUKA simulations to estimate ^41^Ar saturation activity under PET production conditions. Keshavkumar et al. (2017) simulated air activation in a 30 MeV cyclotron vault, demonstrating the dependence of airborne activity on vault geometry and ventilation rate. More recently, Cicoria et al. (2017) performed direct gamma spectrometry to quantify ^41^Ar emissions from a PET cyclotron facility, reporting very low public dose levels.

A foundational contribution to this topic was made by Birattari et al. (1986), who investigated air activation in a 40 MeV proton cyclotron.

In the following years, the average energy of the medical cyclotron has been reduced to a range between 10 and 20 MeV with a current of up to 1 mA in the powerful models, mainly because of economic reasons and the dramatic increase in the beam current.

Currently, more than 1,000 small medical cyclotrons with energies below 20 MeV are installed worldwide, mainly in hospitals, universities, and local commercial plants (IAEA 2025). In addition, about 100 intermediate-energy cyclotrons (20–35 MeV) are typically found in regional commercial facilities and research institutes, while approximately 50 high-energy cyclotrons (>35 MeV) are located in research centers and proton therapy facilities (Goethals and Zimmermann 2020). Most of these cyclotrons are produced by five companies: General Electric Healthcare (10 and 16.5 MeV), IBA (11 and 18 MeV), Siemens (11 MeV), ACSI (19 MeV), and Sumitomo (20 MeV, 12 MeV, and 10 MeV).

The aim of this study is the reassessment of air radioactivation processes, an update of the data concerning the produced radionuclides, and a revision of the parameters originally presented in Birattari et al. (1986), in terms of total activity (Bq) and concentration (Bq L^-1^) at the release point, for cyclotron energies of 10 MeV and 18 MeV of the proton beam. This work also aims to compare the results of three different models: Birattari conic dispersion by Birattari et al. (1986), NCRP 123 Model (NCRP 1996), and HotSpot Model (Homann and Aluzzi 2014).

The choice of 10 MeV and 18 MeV reflects the two most common cyclotron energies used in the production of radionuclides in PET (positron emission tomography). Among the radionuclides listed in Table 1, ^18^F has become the most widely used in medical diagnostics application, primarily due to its physical and chemical characteristics that make it one of the best tracers in PET diagnostics.

**Table 1.** Nuclear reactions and physical yield (Hermanne et al. 2021; Otuka and Takács 2015) of the pharmaceutical radionuclides produced at 10 MeV and 18 MeV proton beam energies.

Nuclear reaction	Physical yield (MBq µA^-1^ h^-1^)	
	10 MeV	18 MeV
^18^O(p,n)^**18**^**F**	2.59 × 10^3^	5.42 × 10^3^
^14^N(p,α)^**11**^**C**	5.07 × 10^3^	1.73 × 10^4^
^16^O(p,α)^**13**^**N**	1.27 × 10^3^	6.95 × 10^3^
^124^Te(p,n)^**124**^**I**	5.17	4.01 × 10^1^
^64^Ni(p,n)^**64**^**Cu**	2.24 × 10^2^	6.76 × 10^2^

In the case of medical cyclotrons, the primary dosimetric concern is related to the production of ^18^F from ^18^O via the (p,n) reaction and the activation of air nuclides (ICRP 2015): when protons are accelerated and interact with ^18^O, they produce neutrons as a byproduct. These neutrons can interact with atoms in the surrounding air, leading to air-activated radionuclides, particularly nitrogen, oxygen, and argon.

The activation of the air is a significant concern because it leads to the contamination of the environment with radioactive isotopes. The exhaust air may contain radioactive nuclides, which could contribute to radiation exposure in areas adjacent to the cyclotron facility or potentially outside the building. Therefore, the modeling of the dose from the exhaust air is of essential importance to ensure that radiation levels in the surrounding environment and workplaces remain within regulatory safety limits.

The parameters used were based on a typical configuration of a medical cyclotron facility located within a hospital. The release point (chimney) was assumed to be approximately 10 m above the sixth floor, corresponding to a total height of about 30 m. Surrounding buildings were assumed to have an average height of 10–15 m, and the nearest off‑site residential receptor was placed at a representative horizontal distance of 100 m from the release point to enable screening‑level comparisons across models.

This configuration represents a general approximation of a standard medical cyclotron facility situated in an urban environment.

Site-specific parameters were deliberately omitted to provide a consistent, model-agnostic screening framework for two reasons: (1) not all models allow detailed customization of meteorological and topographical inputs, and (2) simplifying these characteristics ensures comparability across different modeling approaches.

Omitted factors that can influence near-field concentrations include stack diameter and exit velocity/temperature, building wake and downwash, terrain roughness, mixing height variability, time-resolved meteorology, receptor height and shielding, deposition processes, and chemical speciation of the released gas. Depending on site and meteorology, these effects can either increase or decrease short-range concentrations. The goal was to benchmark models using a single, conservative, simplified set of inputs.

## MATERIAL AND METHODS

The basic equations to calculate the air activation induced by neutrons originated by the proton beams in the target derive from the paper by Birattari et al. (1986).

For cyclotron operating for a time *t* (h) in a room with a volume *V* (cm^3^), the total activity extracted from the vault by a ventilation system during and after the irradiation is given by:


$$A\left(t\right)=\frac{m_{of\;f}a_{s}V}{\lambda +m_{of\;f}}\left[1-e^{-\left(\lambda +m_{on}\right)t}\right]+m_{on}a_{s}V\left[t-\frac{1-e^{-\left(\lambda +m_{on}\right)t}}{\lambda +m_{on}}\right],$$ (1)

where *m*_on_ and *m*_off_ are the air changes per hour respectively in “beam on” and “beam off” mode, $a_{s}$ (Bq cm^-3^) is the saturation activity, and *λ* (h^-1^) is the decay constant of each formed radionuclide. $a_{s}$ is defined as:


$$a_{s}=\frac{\rho \cdot f\cdot N_{A}}{P_{A}}\frac{\lambda \cdot \sum_{i}\frac{\Delta \phi \left(E_{i}\right)}{\Delta E_{i}}\cdot \sigma \left(E_{i}\right)\cdot \Delta E_{i}}{\lambda +m_{i}}.$$ (2)

Here, *σ* (cm^2^) is the reaction cross section, *ρ* (g cm^-3^) the density of target element, *f* the isotopic abundance, *P*_*A*_ (g mol^-1^) the atomic weight of the same element, and *N*_*A*_ is the Avogadro number (6.022 × 10^23^ mol^-1^).

The cross section $\sigma \left(E_{i}\right)$ for all the reactions considered were extracted from the library ENDF/B-VIII.0 (Brown et al. 2018), and we used FLUKA 4-4.1 (Ahdida et al. 2022; Battistoni et al. 2015) and the user interface Flair version 3.3-1 (Vlachoudis 2009) to produce the differential neutron distribution $\Delta \phi \left(E_{i}\right)/\Delta E_{i}$ generated by protons on H_2_^18^O at the energies of 10 MeV and 18 MeV.

For FLUKA, we used the standard defaults described under PRECISIO and JEFF-3.3 as a neutron library with default cut-off values. The differential neutron distribution $\Delta \phi \left(E_{i}\right)/\Delta E_{i}$ was estimated using FLUKA with the USRBDX card. The statistical uncertainties of calculation results were estimated by FLUKA by doing calculations in several batches and computing the standard deviation of the mean: we performed 9 cycles, each consisting of 5 × 10^7^ primaries distributed across 10 spawns, to ensure the uncertainties remained negligible (i.e., below 1%). Our previous paper (Calandrino et al. 2021) presented the energy distribution of secondary neutrons.

The simulation was performed using FLUKA installed on Ubuntu within a Windows 11 Pro machine using the Windows Subsystem for Linux (WSL). The system was equipped with a 13th Gen Intel Core i7-13800H processor running at 2.50 GHz with 64 GB of installed RAM, and the average CPU time used to follow a primary particle was 1.4 × 10^-4^ seconds.

For the evaluation of the released activities, we assumed a significant limited set of reactions (Table 2). In this work, we considered all radionuclides generated in nuclear reactions whose energy thresholds are below the maximum neutron energy [i.e., $\phi_{neutrons}\left(E_{threshold}>E_{neutrons}\right)=0$] and with a half-life of less than 10 y and more than 15 s.

**Table 2.** Possible nuclear reaction in air with half-life (NUDAT 2025) and energy threshold (Wang et al. 2021). In the “Considered nuclear reactions” column, the nuclear reactions utilized for the dosimetric analysis are specified.

Reaction	T_1/2_	Threshold (MeV)	Considered nuclear reactions	
			18 MeV	10 MeV
^16^O(n,p)^16^N	7.5 s	10.2	-	-
^16^O(n,2n)^15^O	2 min	16.7	-	-
^14^N(n,p)^14^C	5730 y	th. energy	-	-
^14^N(n,x)^12^C	12 y	4.3	-	-
^14^N(n,2n)^13^N	10 min	11.3	Yes	-
^40^Ar(n,p)^40^Cl	1.4 min	6.9	Yes	Yes
^40^Ar(n,np)^39^Cl	55 min	12.8	-	-
^40^Ar(n,d)^39^Cl	55 min	10.6	-	-
^40^Ar(n,α)^37^S	5.0 min	2.6	Yes	Yes
^40^Ar(n,γ)^41^Ar	1.83 h	th. energy	Yes	Yes

The reactions within the air–gas mixture and their corresponding relative abundances are summarized in Table 3. All calculations were performed under International Standard Atmosphere (ISA) conditions, as defined by ISO 2533:1975 (ISO 1975). The reactions yielding to ^39^Cl have not been considered due to the coupling of a high value of the energy threshold and a low value of the cross section ($a_{s}=3.7\times 10^{-8}\;Bq\;cm^{-3}$, see Table 4 for a comparison with the other radionuclides).

**Table 3.** Physical and nuclear parameters used for dose calculations of airborne radionuclides. Values include air density (ρ), isotopic abundance (f), atomic weight (P_A_), half-life (T_1/2_), and decay constant (λ) for each selected reaction in air. Air density corresponds to 20°C at 101.325 kPa under standard atmospheric conditions, according to the International Standard Atmosphere (ISA) as defined in ISO 2533:1975 Standard Atmosphere (ISO 1975).

Nuclear reaction	ρ (g cm^-3^)	*f*	*P*_*A*_ (mol^-1^)	*T*_*1/2*_ (h)	λ (h^-1^)
^14^N(n,2n)^**13**^**N**	9.36 × 10^-4^	0.996	14	1.67 × 10^-1^	4.16
^40^Ar(n,p)^**40**^**Cl**	1.12 × 10^-5^	0.996	40	2.33 × 10^-2^	29.70
^40^Ar(n,a)^**37**^**S**	1.12 × 10^-5^	0.996	40	8.33 × 10^-2^	8.32
^40^Ar(n,γ)^**41**^**Ar**	1.12 × 10^-5^	0.996	40	1.83	0.38

**Table 4.** Calculated air saturation activity (a_s_), total activity released (A), and concentration in exhaust air (C) for each radionuclide, assuming a proton beam of 18 MeV and a ventilation scenario of 3 air changes per hour during beam-on time and 10 after irradiation. Results are provided for 1-hour and 5-hour irradiations.

Nuclear reaction	$\sum_{i}\frac{\Delta \phi \left(E_{i}\right)}{\Delta E_{i}}\cdot \sigma \left(E_{i}\right)\cdot \Delta E_{i}$	*a*_s_ (Bq cm^-3^)	*A*(1) (Bq)	*C*(1) (Bq L^-1^)	*A*(5) (Bq)	*C*(5) (Bq L^-1^)
^14^N(n,2n)^**13**^**N**	3.47 × 10^-32^	7.69 × 10^-4^	1.26 × 10^5^	1.26 × 10^-2^	5.87 × 10^5^	1.17 × 10^-2^
^40^Ar(n,p)^**40**^**Cl**	3.96 × 10^-31^	5.75 × 10^-5^	9.09 × 10^3^	9.09 × 10^-4^	4.36 × 10^4^	8.72 × 10^-4^
^40^Ar(n,a)^**37**^**S**	2.94 × 10^-30^	3.45 × 10^-4^	5.66 × 10^4^	5.66 × 10^-3^	2.64 × 10^5^	5.27 × 10^-3^
^40^Ar(n,g)^**41**^**Ar**	4.54 × 10^-30^	8.12 × 10^-5^	1.25 × 10^-4^	1.25 × 10^-3^	6.12 × 10^-4^	1.22 × 10^-3^

Under these hypotheses and using equations defined in the previous paragraph, we have calculated the total amount of released air-activated radionuclides.

After the different amounts of activated radioactivity have been calculated, three different models [Birattari conic plume dispersion by Birattari et al. (1986), NCRP 123 Gaussian plume model (NCRP 1996), and HotSpot general plume model (Homann and Aluzzi 2013)] have been applied to calculate the irradiation and intake dose to neighborhood residential populations at 100 m from the release point. Given that the chimney is assumed to be located so that prevailing winds do not blow toward the nearest houses, assuming an isotropic distribution of wind directions throughout the year can be considered a conservative approach.

Birattari dispersion model considers a conic shape plume with a radius d=0.2×l m where *l* is the distance from the emitting point. The wind speed is assumed $\$v=1\;m\;s^{-1},\$$ and no other meteorological factors will be considered assuming that this could be the worst situation (Sullivan 1982).

It is known (Birattari et al. 1986) that the dose in such a case will be mainly due to gamma and beta external irradiation as a “submersion dose,” any way the intake doses will be calculated to take into account the lung dose for the inhaled mixture (not metabolized). Following the equations by Birattari et al. (1986), the external contribution at the dose D from gamma emitters for 1 h emission is defined by:


$$D_{\gamma }=8.7\times 10^{-3}\cdot \frac{A\cdot \Gamma }{l\cdot v}\;\;\;\;\;\left(mSv\right),$$ (3)

where A is the activity released (MBq), Γ is the specific gamma-ray dose constants (mSv m^-2^ MBq^-1^ h^-1^) (Unger and Trubey 1982), v is the wind speed (i.e., 1 m s^-1^), and l is the distance from the emitting point (i.e., 100 m).

The beta contribution is defined as:


$$D_{\beta }=4.93\times 10^{-4}\cdot \frac{A\cdot \bar{E}}{l^{2}\cdot v}\cdot \frac{{\left(S/\rho \right)}_{t}}{{\left(S/\rho \right)}_{a}}\;\;\;\;\;\left(mSv\right).$$ (4)

A, v and l are defined as above; ${\left(S/\rho \right)}_{t}$ and ${\left(S/\rho \right)}_{a}$ are the mass stopping power for tissue and air (ICRU 1984) and $\bar{E}$ (MeV) is the mean energy for the β particle (Attix 1986).

The intake dose from inhalation is represented by the equation (Birattari et al. 1986; IAEA 2001):


$$D_{int}=\frac{A}{{𝒱}_{P}}\cdot t\cdot R\cdot h\left(g\right)\;\;\;\;\;\left(Sv\right).$$ (5)

Also in this case, A, v, and l are defined as above; ${𝒱}_{P}$ (m^3^) is the volume of the conic shape plume and, in our approximation, it is ${𝒱}_{P}=4.19\times 10^{-2}\cdot l^{3}$; R is the inhalation rate (m^3^ h^-1^); t (h) is the transition time of the plume and calculated as t=(l/v)/3,600 (i.e., t=100 s ≡0.028 h); and h(g) (Sv Bq^-1^) is the inhalation dose coefficient (ICRP 2012).

In our case, we conservatively used R = 1.2 m^3^ h ^-1^, the breathing rate for a reference worker at “light work” (Paquet et al. 2015). For sedentary occupations such as office work, this reference breathing rate is likely to be an overestimate. The dose from intake accounts for at most 0.8% of the total, while the beta dose accounts for 0.4% of the total.

The dispersion model based on NCRP 123 (NCRP 1996) presents a set of conservative, screening-level methodologies for estimating atmospheric dispersion and radiological dose from airborne releases of radionuclides. The model employs a steady-state Gaussian plume formulation, appropriate for continuous point-source emissions under idealized meteorological conditions.

Atmospheric dispersion is calculated using standard Pasquill-Gifford stability classes and associated empirical dispersion parameters (σ_y_, σ_z_), which define the lateral and vertical spread of the plume as a function of downwind distance. The model assumes homogeneous terrain, constant meteorological conditions, and neglects terrain-induced or urban dispersion complexities. Effective plume height is defined by the physical release height and buoyancy parameters when applicable.

The model supports evaluation of key radiological exposure pathways, including inhalation dose, external dose from plume immersion (cloudshine), and external dose from ground deposition (groundshine). Inhalation doses are computed by integrating time-averaged air concentrations with ICRP-derived dose coefficients and assumed breathing rates.

The NCRP 123 methodology is designed to yield conservative (bounding) estimates of a potential off-site dose under limited data conditions and is suitable for preliminary assessments, regulatory screening, and emergency planning scenarios. Due to its simplifying assumptions, particularly the steady-state meteorology and absence of terrain effects, it is not intended for detailed dose reconstruction or site-specific dispersion modeling but rather for fast, protective screening assessments.

Key adjustable parameters of the NCRP 123 model include:

- Source term: Radionuclide type, activity release rate, chemical form, and particle size distribution;
- Meteorological conditions: Wind speed, wind direction, atmospheric stability class, and boundary layer height;
- Release characteristics: Point or area source, release height, plume rise, and deposition mechanisms (dry and wet deposition rates);
- Receptor considerations: Exposure time, inhalation rates, shielding factors, and distances from the release site.

This model assumes a constant concentration in the plume depending on the wind speed, the release rate, and the fraction of the time when the receptor is downwind.

In the simplest case of an isolated point source with the height of the emission (i.e., Chimney point) exceeding 2.5 times the height of the surrounding buildings, therefore neglecting wake effects, the ground level air concentration at the receptor point of the emitted radioactivity can be expressed by the equation:


$$C_{A}=\frac{f\cdot \dot{A}\cdot P}{v}\;\;\;\left(Bqm^{-3}\right),$$ (6)

where f is the fraction of time in which the wind blows in the receptor direction (we assumed f=1 as a precaution in our cases), $\dot{A}$ is the release rate of the radionuclide (Bq s^-1^), v is the wind speed (m s^-1^), and P is the Gaussian diffusion factor related to release height and distance from the receptor, as derived from the equation by Fields and Miller (1980) (m^-2^); in the case of a receptor at a distance of 100 m from the release and a release height of 30 meters, $P=8\times 10^{-5}\;m^{-2}$.

The annual effective dose is given by


$$D=C_{A}\cdot DF\cdot O_{f}\;\;\;\;\;\;\left[Sv\cdot y^{-1}\right],$$ (7)

where $C_{A}$ is the annual average concentration of each radionuclide in air (Bq m^-3^) calculated with eqn (6), DF is the effective dose coefficient (Sv y^-1^ Bq^-1^ m^3^) (NCRP 1996) and $O_{f}$ is the fraction of the year for which the hypothetical critical group member is exposed to this particular pathway (we assumed $O_{f}=1$ as a precaution in our cases).

Internal exposure from ingestion or inhalation of ^37^S, ^40^Cl, and ^13^N was not considered because these radionuclides have very short physical half-lives (minutes), resulting in negligible systemic uptake before decay. This is consistent with the absence of intake coefficients for these nuclides in ICRP 119 (International Commission on Radiological Protection, 2012), which otherwise provide age-dependent values for most environmentally relevant radionuclides. For ^41^Ar, the internal dose from inhalation is also negligible compared to external exposure from cloud immersion, which dominates the effective dose for noble gases. To quantify this pathway, we used EPA FGR‑15 (US EPA 2025) air-submersion dose-rate coefficients (Table 4.3), which incorporate ICRP 107 (ICRP 2008) decay data and age-specific phantoms, ensuring consistency with current US federal guidelines. For transparency, the corresponding ICRP 119 Annex C adult coefficient (5.3 × 10^-9^ Sv d^-1^ Bq^-1^ m^3^) is numerically consistent with FGR‑15 values. This approach aligns with modern regulatory practices and avoids reliance on outdated screening factors in NCRP 123, which were based on earlier dosimetric models and do not include these activation products.

HotSpot (Homann and Aluzzi 2014) is a suite of Windows-based computational tools developed by Lawrence Livermore National Laboratory to facilitate rapid assessment of radiological releases into the atmosphere. These codes are employed by health physicists, emergency responders, and radiation protection professionals for evaluating the potential radiological consequences of nuclear incidents, including accidental releases and radiological emergencies.

The primary objective of HotSpot is to provide a scientifically credible yet user-friendly platform that enables swift estimation of radiation doses and contamination spread within short to moderate distances — typically up to several tens of kilometers from the release site. It leverages Gaussian plume and puff dispersion models to simulate the atmospheric transport, diffusion, and deposition of radionuclides following an airborne release.

It is possible to customize key parameters to tailor simulations to specific scenarios, including:

- Source Term: Radionuclide type, activity level, particle size, and release height;
- Meteorological Conditions: Wind speed, wind direction, atmospheric stability class, and mixing layer height;
- Release Characteristics: Continuous or instantaneous release, duration, and deposition properties (wet/dry deposition rates);
- Geographical Inputs: Terrain roughness, receptor locations, and distance from the release point.

The codes compute external gamma dose rates from the passing plume, inhalation doses based on airborne concentrations, and groundshine doses due to deposited material.

HotSpot generates spatial dose distributions, plume contours, and population dose estimates, which can be used to inform protective action decisions such as evacuation or sheltering. It also includes modules for estimating acute health effects, including early fatalities and injuries based on the calculated dose levels.

While HotSpot offers rapid and accessible assessments through an intuitive graphical user interface, it is important to note that its underlying Gaussian models represent simplified atmospheric dispersion and may not capture complex meteorological phenomena or terrain effects inherent in real-world scenarios. Consequently, HotSpot is most suitable for initial screening, emergency response, and training purposes rather than detailed environmental impact analyses.

HotSpot has a set of variables with a higher level of complexity than the previous models; 12 different models of the dispersion are presented in the first menu of the software. For our purposes, we used the “general plume model.”

In a general calculation of the dose, the meteorological site conditions can be neglected, assuming a conservative wind speed of 1 m s^-1^.

These are the parameters of the model:

- Topography of the surroundings: city;
- Material At Risk (MAR) is the total quantity of the radionuclide involved in the release scenario: the released activity during 1 wk;
- Damage Ratio (DR) is the fraction of the MAR in the release scenario: set =1;
- LeakPath Factor (LPF) is the unfiltered fraction: set =1;
- AiRborne Fraction (ARF) is the fraction of the MAR released in atmosphere: set=1;
- Emission height: 30 m;
- Distance receptor emission point: 100 m; and
- Emission duration: 30 h.

This model calculates and outputs total effective dose (sum of all radiation doses — internal and external — a person receives) estimates for individuals for each isotope in Sv for 1 wk.

We made all the previously presented evaluations for 10 MeV and 18 MeV proton beams.

In this first-level screening analysis, uncertainties were not considered. This approach was justified by the fact that the underlying FLUKA simulations, used to calculate activities according to the NCRP 123 and “Birattari” (Simple Dispersion Model) models, were specifically designed to minimize statistical uncertainties, maintaining them below 1%.

## RESULTS AND DISCUSSION

For our calculations, we have considered two scenarios. The first assumes an air exchange rate of 3 during the operation of the cyclotron (i.e., $m_{on}=3$); that is, the minimum number of exchanges required to maintain negative pressure inside the room (i.e., a relative internal pressure of -50/150 Pa) where the cyclotron is installed (room volume is 50 m^3^). After irradiation, the air exchange rate is increased ($m_{of\;f}=10$) to speed up the removal of any residual radioactive isotopes and to ensure that any contamination is quickly diluted and vented out of the room.

The second scenario assumes a constant airflow of 10 air exchanges per hour (i.e., $m_{on}=m_{of\;f}=10$), as suggested by UNI 10491:1995 (UNI 1995).

Using this information and the data from Table 3, eqn (1), and eqn (2), we calculated the values of A(1) and A(5) (Table 4 and Table 5) for the first scenario, where A(1) is the total activity released (Bq) for a 1‑h target irradiation followed by post‑irradiation ventilation, and A(5) is the analogous 5‑h case. These are the two building blocks we combine linearly to reproduce a typical weekly workload mix (eqn 8).

**Table 5.** Same as Table 4, but for a 10 MeV proton beam.

Nuclear reaction	$\sum_{i}\frac{\Delta \phi \left(E_{i}\right)}{\Delta E_{i}}\cdot \sigma \left(E_{i}\right)\cdot \Delta E_{i}$	*a*_s_ (Bq cm^-3^)	*A*(1) (Bq)	*C*(1) (Bq L^-1^)	*A*(5) (Bq)	*C*(5) (Bq L^-1^)
^40^Ar(n,p)^**40**^**Cl**	5.50 × 10^-37^	7.98 × 10^-11^	1.26 × 10^-2^	1.26 × 10^-9^	6.05 × 10^-2^	1.21 × 10^-9^
^40^Ar(n,a)^**37**^**S**	1.98 × 10^-33^	2.32 × 10^-7^	3.81 × 10^1^	3.81 × 10^-6^	1.77E×10^2^	3.55 × 10^-6^
^40^Ar(n,g)^**41**^**Ar**	9.98 × 10^-31^	1.79×. 10^-5^	2.75 × 10^3^	2.75 × 10^-4^	1.35 × 10^4^	2.69 × 10^-4^

The airflow rate of the system into which the exhaust from the room housing the cyclotron is discharged is 10,000 m^3^ h^-1^, which yields the stack concentrations C(1) and C(5). The values of C(1) and C(5) reported in Table 4 and Table 5 indicate that the concentration of all radionuclides is below the legal threshold of 1 Bq g^−1^ (in air, 1 Bq L^-1^ ≈ 1 Bq g^−1^) (Council of the European Union 2014; Repubblica Italiana 2020).

Once the values for A(1) and A(5) are obtained, it is possible to calculate the total activity released per year by a simple linear combination of these two.

In this work, we assume a total weekly workload of 3,000 µA h wk^-1^ at the current of 100 µA; that is 30 h of beam time assuming, on average, 2 irradiations of 5 h and 20 irradiations of 1 h each:


$$A_{week}=2\times A\left(5\right)+20\times A\left(1\right).$$ (8)

Weekly fluctuations in the number or duration of irradiations are expected, but they are averaged over the year and therefore do not significantly affect the overall evaluation. The yearly released activity will be 50 times the total of the eqn (8).

We want to calculate the population doses, for people living 24 h d^-1^ at the distance of 100 m from the chimney, using the three different models introduced in the previous section.

The comparison of the results obtained by the three models is presented for the two energies of protons in Table 6 and Table 7.

**Table 6.** Estimated annual effective dose to a member of the public located 100 meters downwind from the release point. Doses are calculated for an 18 MeV proton beam and 3 air changes per hour ventilation scenario, using three dispersion models.

Radionuclide	Total released radioactivity MBq y^-1^	Dose µSv·y^-1^		
		Birattari	NCRP 123	HotSpot
^13^N	1.85 × 10^2^	3.87 × 10^-3^	5.40 × 10^-4^	1.40 × 10^-3^
^40^Cl	1.34 × 10^1^	1.80 × 10^-3^	1.80 × 10^-4^	2.40 × 10^-4^
^37^S	8.30 × 10^1^	1.33 × 10^-2^	8.18 × 10^-4^	1.85 × 10^-3^
^41^Ar	1.86 × 10^1^	4.52 × 10^-4^	7.33 × 10^-5^	2.00 × 10^-4^
**TOTAL DOSES**		1.95 × 10^-2^	1.61 × 10^-3^	3.69 × 10^-3^

**Table 7.** Same as Table 6, but for a 10 MeV proton beam. Note the absence of ^13^N due to reaction threshold considerations.

Radionuclide	Total released radioactivity MBq y^-1^	Dose µSv·y^-1^		
		Birattari	NCRP 123	HotSpot
^40^Cl	1.87 × 10^-5^	1.79 × 10^-9^	2.50 × 10^-10^	3.35 × 10^-10^
^37^S	5.58 × 10^-2^	2.74 × 10^-6^	5.50 × 10^-7^	1.25 × 10^-6^
^41^Ar	4.09	9.62 × 10^-5^	1.61 × 10^-5^	4.45 × 10^-5^
**TOTAL DOSES**		9.89 × 10^-5^	1.67 × 10^-5^	4.58 × 10^-5^

The second scenario hypothesizes that the air exchange rate is 10 per hour during operation (i.e., $m_{on}=10$) and 10 per hour after irradiation (i.e., $m_{of\;f}=10$).

We calculated the values of A(1) and A(5) (Table 8 and Table 9) and the comparison of the results obtained by the three models is presented for the two proton energies in Table 10 and Table 11.

**Table 8.** Calculated air saturation activity (a_s_), total activity released (A), and concentration in exhaust air (C) for each radionuclide, assuming aroton beam of 18 MeV and a ventilation scenario of 10 air changes per hour during beam-on time and 10 after irradiation. Results are provided for 1-hour and 5-hour irradiations.

Nuclear reaction	*a*_s_ (Bq cm^-3^)	*A*(1) (Bq)	*C*(1) (Bq L^-1^)	*A*(5) (Bq)	*C*(5) (Bq L^-1^)
^14^N(n,2n)^**13**^**N**	3.89 × 10^-4^	1.94 × 10^5^	1.94 × 10^-2^	9.71 × 10^5^	3.89 × 10^-4^
^40^Ar(n,p)^**40**^**Cl**	4.74 × 10^-5^	2.37 × 10^4^	2.37 × 10^-3^	1.18 × 10^5^	4.74 × 10^-5^
^40^Ar(n,a)^**37**^**S**	2.13 × 10^-4^	1.07 × 10^5^	1.07 × 10^-2^	5.33 × 10^5^	2.13 × 10^-4^
^40^Ar(n,g)^**41**^**Ar**	2.64 × 10^-5^	1.32 × 10^4^	1.32 × 10^-3^	6.61 × 10^4^	2.64 × 10^-5^

**Table 9.** Same as Table 8, but for a 10 MeV proton beam.

Nuclear reaction	*a*_s_ (Bq cm^-3^)	*A*(1) (Bq)	*C*(1) (Bq L^-1^)	*A*(5) (Bq)	*C*(5) (Bq L^-1^)
^40^Ar(n,p)^**40**^**Cl**	6.58 × 10^-11^	3.29 × 10^-2^	3.29 × 10^-9^	1.64 × 10^-1^	3.29 × 10^-9^
^40^Ar(n,a)^**37**^**S**	1.43 × 10^-7^	7.17 × 10^1^	7.17 × 10^-6^	3.58 × 10^2^	7.17 × 10^-6^
^40^Ar(n,g)^**41**^**Ar**	5.82 × 10^-6^	2.91 × 10^3^	2.91 × 10^-4^	1.45 × 10^4^	2.91 × 10^-4^

**Table 10.** Estimated annual effective dose at 100 meters for 18 MeV beam and 10 air changes per hour ventilation. Compared across three dispersion models.

Radionuclide	Total released radioactivity MBq y^-1^	Dose µSv y^-1^		
		Birattari	NCRP 123	HotSpot
^13^N	2.91 × 10^2^	6.10 × 10^-3^	8.50 × 10^-4^	2.20 × 10^-3^
^40^Cl	3.55 × 10^1^	4.76 × 10^-3^	4.75 × 10^-4^	6.36 × 10^-4^
^37^S	1.60 × 10^2^	2.57 × 10^-2^	1.58 × 10^-3^	3.56 × 10^-3^
^41^Ar	1.98 × 10^1^	4.82 × 10^-4^	7.82 × 10^-5^	2.13 × 10^-4^
**TOTAL DOSES**		3.70 × 10^-2^	2.98 × 10^-3^	6.61 × 10^-3^

**Table 11.** Same as Table 10, but for 10 MeV beam. Dose values remain significantly below 1 µSv y^-1^ for all radionuclides and models.

Radionuclide	Total released radioactivity MBq y^-1^	Dose µSv y^-1^		
		Birattari	NCRP 123	HotSpot
^40^Cl	4.93 × 10^-5^	4.73 × 10^-9^	6.60 × 10^-10^	8.69 × 10^-10^
^37^S	1.08 × 10^-1^	5.28 × 10^-6^	1.06 × 10^-6^	2.40 × 10^-7^
^41^Ar	4.36	1.03 × 10^-4^	1.72 × 10^-5^	4.73 × 10^-5^
**TOTAL DOSES**		1.08 × 10^-4^	1.83 × 10^-5^	4.97 × 10^-5^

The dose estimates reported in Table 6, Table 7, Table 10, and Table 11 illustrate the behavior of the three dispersion models under varying proton energies and ventilation regimes. In all cases, the Birattari conic plume model yields the highest effective dose values, reflecting its conservative assumptions about localized atmospheric dispersion and limited dilution of the radioactive plume. HotSpot generally produces intermediate results, thanks to its more detailed parameterization of release conditions and environmental settings, while NCRP 123 systematically produces the lowest dose estimates due to its reliance on a semi-infinite cloud assumption, which tends to underestimate near-field exposures. For very short-lived nuclides like ^40^Cl, the balance between in-plume decay and advection makes near-field immersion doses highly sensitive to wind speed and plume geometry, explaining the greater model variability compared with ^13^N or ^41^Ar. By contrast, ^37^S combines a short half-life with strong high-energy γ emission (3.1 MeV), resulting in elevated cloudshine per Bq at the receptor. As expected, the total population doses at 10 MeV are significantly lower than those at 18 MeV, owing to reduced neutron production and activation cross-sections. Interestingly, while increased ventilation (10 air changes per hour) enhances air removal, it also reduces the decay time available for short-lived isotopes within the vault, leading to higher released activities and, consequently, slightly higher effective doses, especially for isotopes like ^13^N. However, all predicted doses remain well below regulatory concern thresholds, reinforcing the low radiological impact of routine airborne releases in medical cyclotron operations.

Our results are consistent with those reported in previous studies. For example, the ^41^Ar saturation activity and public dose values we obtained agree with those measured by Cicoria et al. (Cicoria et al. 2017), who reported annual doses below 0.2 µSv y^-1^. Similarly, the trends observed in our FLUKA simulations, especially regarding the influence of ventilation and beam energy, are in line with the findings of Infantino et al. (2015) and Keshavkumar et al. (2017). This agreement reinforces the validity of our modeling approach and supports the conclusion that, under typical operational scenarios, airborne emissions from medical cyclotrons contribute negligibly to public radiation exposure.

However, releases from synthesis/hot‑cell processes can dominate airborne activity in busy facilities, potentially yielding off‑site doses that are 3-4 orders of magnitude higher than vault air activation if not properly trapped/filtered (Calandrino et al. 2009); therefore, robust hot‑cell off‑gas recovery is mandatory.

## CONCLUSION

This study provides a comprehensive assessment of air activation in medical cyclotrons operating at 10 MeV and 18 MeV proton beam energies. By applying well-established models, including the Birattari conic plume dispersion model, the NCRP 123 model, and the HotSpot model, we evaluated the release and dispersion of activated air radionuclides, estimating their potential radiological impact on the surrounding environment.

The presented results demonstrate that also in an extreme precautionary set of hypotheses, with the wind speed as low as reasonable and the wind direction always toward the target point, the contribution of the dose to the population residing nearby could be estimated in the order of a fraction of µSv y^-1^ in the case of 18 MeV protons and at least two orders of magnitude less for the 10 MeV protons beam. Moreover, in all the models we have maintained the assumption of a release point in a chimney, with a position and a height capable of skipping the fallout of the plume on the same building or around the facility itself.

Comparing the three dispersion models, we found that HotSpot, due to its complexity, provides the most detailed dose estimation, while the NCRP 123 model yields the lowest dose estimates among the three models examined. It remains a widely used regulatory tool due to its well-established methodology, transparent parameter set, and suitability for generic, long-term environmental assessments. However, its simplifying assumptions, particularly the use of a semi-infinite cloud, can lead to underestimations in near-field scenarios or when emissions originate from short stacks. The Birattari conic plume dispersion model provides a conservative estimate of exposure: it is well known that the Birattari conic‑plume assumption (Birattari et al. 1986) yields the most conservative (highest) near‑field dose estimates among the models considered, especially at short source‑to‑receptor distances (Balestreri et al. 2018).

As expected, the slowing down of the air change rate during the beam time minimizes the environmental dispersion of the activated radionuclides (Table 6 vs. Table 10 and Table 7 vs. Table 11). Because a safe negative pressure differential (≈ -50 to -150 Pa) must be maintained in the cyclotron vault during bombardment to prevent leakage into adjacent areas, the ventilation rate cannot be reduced to zero. Each facility should verify the minimum rate needed to preserve this negative pressure safely.

The decrease in the ventilation speed during beam-on time is commonly accepted to reduce the outlet of contaminated air outside the bunker, but the gain in dose saving is entirely negligible when we compare the results obtained with a stable ventilation rate.

We recommend keeping the ventilation rate at the defined value and using an emergency regimen where the lowest change rate can be achieved to maintain the internal negative pressure, in the case of leaks from targets or valves during bombardment or isotopes transfer.

*Acknowledgments* — This paper has originated from the groundbreaking paper by Professor Claudio Birattari (1940-2017) who was in charge of the Health Physics School of University of Milan for several years.

At the beginning of the 1980s, some of us attended the core courses he held at the School. We are all pleased to dedicate this work, an update of his 1986 milestone, to his memory with a lot of gratitude for his efforts in building a community of professionals in the field of medical physics. Thanks, Claudio.
